# Efficient Thermal-Stress Coupling Design of Chiplet-Based System with Coaxial TSV Array

**DOI:** 10.3390/mi14081493

**Published:** 2023-07-25

**Authors:** Xianglong Wang, Jiaming Su, Dongdong Chen, Di Li, Gaoliang Li, Yintang Yang

**Affiliations:** 1 School of Microelectronics, Xidian University, Xi’an 710071, China; xlwang_312@stu.xidian.edu.cn (X.W.); ytyang@xidian.edu.cn (Y.Y.); 2 CCTEG Ecological Environment Technology Co., Ltd., Beijing 100013, China; hbzzrzyj@163.com

**Keywords:** coaxial through silicon via, Chiplet-based system, interconnect structure, thermal-stress coupling

## Abstract

In this research, an efficient thermal-stress coupling design method for a Chiplet-based system with a coaxial through silicon via (CTSV) array is developed by combining the support vector machine (SVM) model and particle swarm optimization algorithm with linear decreasing inertia weight (PSO-LDIW). The complex and irregular relationship between the structural parameters and critical indexes is analyzed by finite element simulation. According to the simulation data, the SVM model is adopted to characterize the relationship between structural parameters and critical indexes of the CTSV array. Based on the desired critical indexes of the CTSV array, the multi-objective evaluation function is established. Afterwards, the structural parameters of the CTSV array are optimized through the PSO-LDIW algorithm. Finally, the effectiveness of the developed method is verified by the finite element simulation. The simulated peak temperature, peak stress of the Chiplet-based system, and peak stress of the copper column (306.16 K, 28.48 MPa, and 25.76 MPa) well agree with the desired targets (310 K, 30 MPa, and 25 MPa). Therefore, the developed thermal-stress coupling design method can effectively design CTSV arrays for manufacturing high-performance interconnect structures applied in Chiplet-based systems.

## 1. Introduction

With the development of semiconductor manufacturing processes, Chiplet-based systems have been widely investigated to achieve the continuation of Moore’s law [1,2,3]. Due to the advantages of miniaturization, high performance, and low cost, Chiplet-based systems have been applied in computing systems and processing-in-memory systems [4,5,6]. Through silicon via (TSV) is a key technology to achieve vertical interconnection between different dies in Chiplet-based systems [7,8,9,10,11]. In order to satisfy the requirements of various applications, cylindrical, tapered, and coaxial TSVs (CTSVs) have been proposed [12,13,14]. Due to the advantage of self-shielding, the CTSV can effectively suppress the signal crosstalk and minimize the signal transmission delay, so it has been widely applied in the integrated system [15,16].

The CTSV has been systematically investigated by many researchers in recent years [17,18,19]. Adamshick et al. [20] characterized the thermomechanical behavior of CTSV based on the micro-Raman spectroscopy technique. Yang et al. [21] proposed a new CTSV structure that offers more shielding and less coupling with adjacent structures compared with conventional CTSV. Based on the finite element method (FEM), Qian et al. [22] proposed a distributed transmission line model of silicon-core CTSV. The simulation results observed that the thickness of the plated Cu and isolation dielectric resolved the electrical performance of the silicon-core CTSV. Qiu et al. [23] performed electrical-thermal co-simulation with consideration of the temperature dependence of the metal-oxide-semiconductor effect for CTSV by using equivalent electrical and thermal circuit models. Wang et al. [24] proposed an analytical model for the thermal stress caused by the CTSV and gave guidelines for the design of the CTSV. Dong et al. [25] presented an accurate TSV thermal mechanical stress analytical model, which is verified by FEM. However, the Chiplet-based system with the CTSV array is rarely reported. Therefore, it is valuable to investigate the thermal-stress coupling design of a Chiplet-based system with a CTSV array.

The FEM has the advantages of high computational accuracy, good mesh adaptability, and easy handling of irregular geometric areas, but its disadvantages are that it is time-consuming and inefficient for large-scale simulation [26]. In recent years, data-driven models, such as the support vector machine (SVM) model, neural networks, machine learning, etc., have been applied in medicine, information, industry, etc. Due to their advantages in adaptivity, simplicity, fault tolerance, and robustness, neural networks have been widely used in image processing [27,28], model-predicted control [29,30] data extraction [31,32], etc. The deep neural network has been applied in large-scale integrated systems to describe complex relationships [33]. There are many regression models, such as the linear regression model, the random forest model, the SVM model, etc. Due to its high accuracy, generalization, and non-linear characterization, the SVM model has been used in complex engineering problems such as regression estimation of data, global sensitivity evaluation, and estimation of alloy flow characteristics [34,35,36,37]. The SVM model is good at dealing with linearly indivisible sample data and can effectively avoid data overfitting. In addition, the disadvantages of FEM are that it is time-consuming and inefficient for large-scale simulation, so SVM can be used to replace the FEM model to improve the simulation’s efficiency. At the same time, the particle swarm optimization algorithm with linear decreasing inertia weight (PSO-LDIW) has been widely used in the optimization of electronic components [28,38,39]. Thus, the PSO algorithm can be used to optimize the structural parameters of the CTSV array to design a Chiplet-based system with high thermal stress performance.

In this research, an efficient thermal-stress coupling design method for Chiplet-based systems with CTSV arrays is developed based on the SVM model and PSO-LDIW algorithm. And the highlights can be summarized as follows:

(1) The complex relationship between structural parameters and critical indexes of the CTSV array is analyzed by FEM simulation.
(2) The SVM model is established to rapidly characterize the complex relationship between structural parameters and critical indexes of the CTSV array.
(3) The efficient thermal-stress coupling design method is developed under the framework of the PSO algorithm to control the critical indexes of the CTSV array.

The remainder of this paper is arranged as follows: In Section 2, the modeling and simulation of the thermal-stress coupling CTSV array are presented. In Section 3, the thermal-stress coupling design method for Chiplet-based systems with CTSV arrays is presented. The conclusions are finally given in Section 4.

## 2. Model and Simulation of Thermal-Stress Coupling CTSV Array

In this research, the FEM model of a Chiplet-based system with a CTSV array is established based on COMSOL software 6.0. In addition, the relationship between structural parameters and critical indexes of the CTSV array is analyzed by the FEM simulation results according to the orthogonal design experiment.

### 2.1. Thermal-Stress Coupling CTSV Array Model

**Figure 1:**
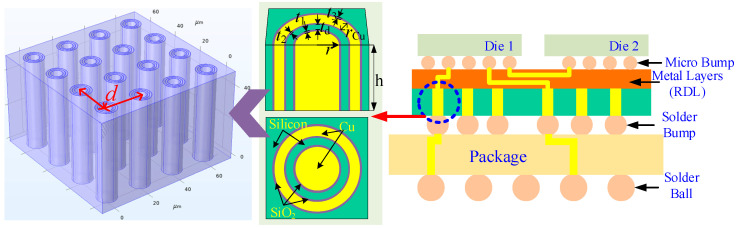
Schematic of CTSV in Chiplet-based system.

In this research, a 4 × 4 CTSV array model applied in Chiplet-based systems is established, and the schematic of CTSV in Chiplet-based systems is shown in Figure 1. The CTSV unit is composed of a metal column, a coaxial metal ring, and a dielectric filling layer. The metal column is used to transmit the electrical signal, while the coaxial metal ring is used as a shielding layer for signal interference and noise. The dielectric filling layer is located between the signal layer and the shielding layer. Because of its low resistivity and electrical conductivity [40], copper is commonly used as the TSV-filled material, so the metal column and coaxial metal ring are filled with copper. In addition, the basic properties of the materials used in the FEM model are shown in Table 1. The DC excitation is loaded above the copper column, and the value is 8 × 10^10^ A/m^2^. The electric potential is 0.1 V. The fixed constraint is set around the FEM model. The ambient temperature is 293.15 K. The heat transfer coefficient of convective heat flux is 10 W/(m^2^·K).

### 2.2. Simulation and Results

**Figure 2:**
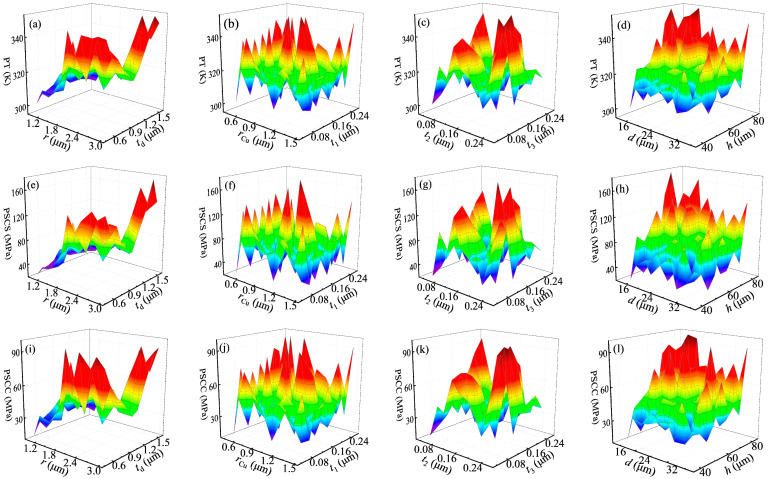
Simulation results based on the orthogonal experiments: (**a**–**d**): peak temperature; (**e**–**h**): peak stress of the Chiplet-based system; (**i**–**l**): peak stress of copper column.

In this research, the structural parameters of the CTSV array are the radius of the copper column (*r*), the thickness of the dielectric layer (*t*_d_), the thickness of the coaxial copper ring (*r*_Cu_), the thickness of the oxide layer between the copper column and shielding layer (*t*_1_), the thickness of oxide layer between the coaxial copper ring and shielding layer (*t*_2_), the thickness of the oxide layer in the copper ring (*t*_3_), the distance between CTSV units (*d*), and the height of CTSV units (*h*). The critical indexes of the CTSV array are the peak temperature (PT), peak stress of the Chiplet-based system (PSCS), and copper column (PSCC). The range of *r* is [1, 3] μm. The ranges of *t*_d_ and *r*_Cu_ are [0.5, 1.5] μm. The ranges of *t*_1_, *t*_2_, and *t*_3_ are [0.05, 0.25] μm. The ranges of *d* and *h* are [15, 35] and [40, 80] μm, respectively. The design of the experiment used in this study is an orthogonal experiment. The orthogonal experiment is utilized in the finite element simulation, which includes 8-factors and 9-levels. The orthogonal experiment is shown in Table 2. The number of the FEM simulation is 81, which is obtained by the IBM SPSS Statistics software 24. In the software, the orthogonal plan can be obtained after entering the factors and levels. According to the scheme of orthogonal design, if the level is 9, the number of orthogonal experiments is 81. The simulation results of the CTSV array are shown in Figure 2, based on the orthogonal experiment. These figures are plotted by the Origin 2021 software. According to the orthogonal design, the critical indexes of the CTSV array vary with the structural parameters. The changes in PT with the structural parameters are shown in Figure 2a–d. The range of PT is [300.88, 352.06] K. From Figure 2a, it can be seen that The PT is gradually decreasing with the increase of *r.* The PT is first increased, then decreased, and finally increased with the increase in *t*_d_. From Figure 2b,c, the PT is gradually increased and then decreased with the increase of *r*_Cu_. The PT is gradually increased, then decreased with the increase of *t*_1_, *t*_2_, and *t*_3._ From Figure 2d, the PT first decreases with the increase of *d* and then gradually increases with the increase in *h*. The changes in PSCS and PSCC with the structural parameters are shown in Figure 2e–l. The ranges of PSCS and PSCC are [21.05, 176.74] and [13.30, 97.30] MPa, respectively. From Figure 2e,i, it can be seen that the PSCS and PSCC are first increased and then decreased with the increase of *r*. The PSCS and PSCC are first increased, then decreased, and gradually increased with the increase in *t*_d_. From Figure 2f,g,j,k, the PSCS and PSCC are increased and then decreased with the increase of *r*_Cu_. The PSCS and PSCC are gradually increasing with the decrease in *t*_1_, *t*_2_, and *t*_3_. From Figure 2h,l, it can be seen that the PSCS and PSCC are increased with the decrease of *d*. The PSCS and PSCC decrease with the increase in *h*. Due to the fact that these eight structural parameters collectively affect the critical index, the above analysis is only a discussion of the approximate variation of the critical indexes with the structural parameters. Undoubtedly, the thermal and stress distributions vary with the structural parameters, and the relationship between the structural parameters and critical indexes of the CTSV array is complex and irregular.

## 3. Efficient Thermal-Stress Coupling Design for CTSV Array

**Figure 3:**
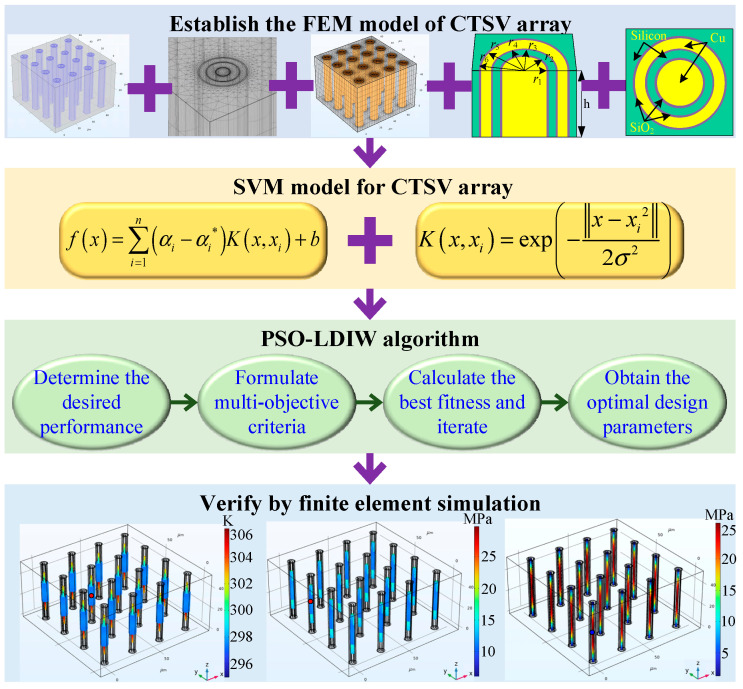
Flowchart of the developed method.

In this research, an efficient thermal-stress coupling design method for Chiplet-based systems with CTSV arrays is developed by combing the PSO-LDIW algorithm with the SVM model. The flowchart of the developed method is shown in Figure 3, which can be divided into four steps. Firstly, the 4 × 4 CTSV array applied in Chiplet-based systems is established based on COMSOL Multiphysics 6.0. Secondly, the SVM model is established based on the database obtained by orthogonal experiments. Thirdly, the structural parameters of the CTSV array are optimized by the PSO-LDIW algorithm. Finally, the effectiveness of the optimized structural parameters is verified by finite element simulation. The details of the thermal-stress coupling design for the CTSV array are presented as follows:

### 3.1. Critical Database Model and Optimization Algorithm

**Figure 4:**
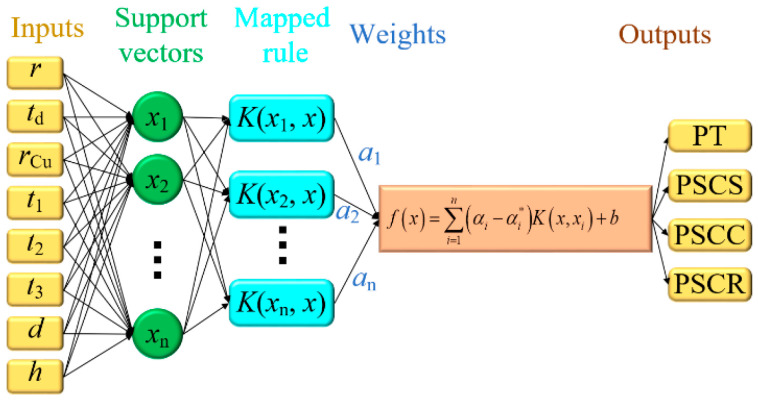
Architecture of support vector machine model.

Based on the simulation results of the orthogonal experiment, the complex relationship between structural parameters and critical indexes of the CTSV array is characterized by the SVM model. The architecture of the SVM model is shown in Figure 4. The capacity constant (*C*), insensitive parameter (*e*), and kernel function ($K(x_{i},x_{j})$) are the key parameters of the SVM model. The inputs of the SVM model are the structural parameters (*r*, *t*_d_, *r*_Cu_, *t*_1_, *t*_2_, *t*_3_, *d*, *h*). The outputs are the critical indexes (PT, PSCS, and PSCC). Three multi-input, single-output SVM models are established. The appropriate values of *C* are 100,000, 100,000, and 100,000. The values of *e* are 0.1, 1, and 1. The Gauss kernel function is chosen as the kernel function. The validity of the SVM model is tested by taking the data from the FEM simulation results as test samples. The errors of the three SVM models are 3.31 × 10^−7^, 3.55 × 10^−6^ and 3.02 × 10^−6^, respectively, which indicates that the SVM model has high accuracy. When the testing is applied with the data from a training dataset, the error remains at a very low level. The testing data for the established SVM model is shown in Table 3. Obviously, the maximum relative errors for the critical indexes are all less than 5.05%. In the fixed ranges, the higher the accuracy of the established SVM model, the more beneficial the precise design of the CTSV array is.

In addition, the critical indexes of the thermal-stress coupling CTSV array model are the PT, PSCS, and PSCC. The multi-objective evaluation function *F* has been constructed:

(1) $$F=\alpha J_{\mathrm{PT}}{}^{2}+\beta J_{\mathrm{PSCS}}{}^{2}+\gamma J_{\mathrm{PSCC}}{}^{2}$$

where *J*_PT_, *J*_PSCS_, and *J*_PSCC_ represent the optimization criteria for PT, PSCS, and PSCC, respectively. The *α*, *β*, and *γ* are the weight coefficients, and their values are 1/3, respectively. To prevent the influence of magnitude, the optimization criterion after normalization for PT, PSCS, and PSCC can be characterized as

(2) $$J_{\mathrm{PT}}=\frac{PT-PT_{\mathrm{des}}}{PT_{\max}-PT_{\min}}$$

(3) $$J_{\mathrm{PSCS}}=\frac{PSCS-PSCS_{\mathrm{des}}}{PSCS_{\max}-PSCS_{\min}}$$

(4) $$J_{\mathrm{PSCC}}=\frac{PSCC-PSCC_{\mathrm{des}}}{PSCC_{\max}-PSCC_{\min}}$$

where *PT*_des_, *PSCS*_des_, and *PSCC*_des_ represent the desired *PT*, *PSCS*, and *PSCC*, respectively. *PT*_max_, *PT*_min_, *PSCS*_max_, *PSCS*_min_, *PSCC*_max_, and *PSCC*_min_ are the maximum and minimum of PT, PSCS, and PSCC, respectively. When *J*_PT_, *J*_PSCS_, and *J*_PSCC_ are all less than 0 or the algorithm reaches the maximum number of iterations, the optimization algorithm is stopped. The structural parameters of the CTSV array are optimized by the PSO-LDIW algorithm, and the pseudocode is presented in Algorithm 1. The parameters used in the PSO-LDIW algorithm are shown in Table 4. The desired target values of PT, PSCS, and PSCC are determined based on extensive simulation analysis. These desired target values are as small as possible. In addition, the multi-objective evaluation function is established based on the desired target values. And then, the PSO-LDIW algorithm is used to optimize the structural parameters of the CTSV array so that the critical indexes can reach the desired target values as close as possible.
**Algorithm 1** Pseudocode of the efficient thermal-stress coupling design of CTSV array under the framework of PSO algorithm1Initialize population2for *t* = 1:maximum generation3  Initialize local and global best particles (*p*_i_ and *p*_g_)4  for *i* = 1:population size5   for *d* = 1:dimension6    *v_i,d_*(*t* + 1) = *w*(*t*)*v_i_*_,*d*_(*t*) + *c*_1_*r*_1_(*p_i_*-*x_i_*_,*d*_(*t*)) + *c*_2_*r*_2_(*p*_g_-*x_i_*_,*d*_(*t*));7      if *v_i_*_,*d*_(*t* + 1) > *v*_max_ then *v_i_*_,*d*_(*t* + 1) = *v*_max_;8          else if *v_i_*_,*d*_(*t* + 1) < *v*_min_ then *v_i_*_,*d*_(*t* + 1) = *v*_min_;9      end10        *x_i_*_,*d*_(*t* + 1) = *x_i_*_,*d*_(*t*) + *v_i_*_,*d*_(*t*+1);11        if *x_i_*_,*d*_(*t* + 1) > *x*_max_ then *x_i_*_,*d*_(*t* + 1) = *x*_max_;12          else if *x_i_*_,*d*_(*t* + 1) < *x*_min_ then *x_i_*_,*d*_(*t* + 1) = *x*_min_;13        end14     end15     if *f*(*x_i_*_,*d*_(*t*)) < *f*(*p_i_*(*t*)) then *p_i_*(*t*) = *x_i_*_,*d*_(*t*);16     end17  end18  *f*(*p*_g_(*t*)) < min*_i_*(*f*(*p_i_*(*t*)));19  *w*(*t*) = [(*t*_max_ − *t*)/*t*_max_](*w*_max_ − *w*_min_) + *w*_min_20  *P*_best_(*t*) = *f*(*p*_g_(*t*))21  if *P*_best_(*t*) < eps then break;22  end23End*x*_i_ and *v*_i_ represent the position and velocity of the *i*th particle; *p*_i_ is the best previous position of the *i*th particle, and *p*_g_ is the best previous position of all particles; *t*_max_ is the maximum number of iterations; *w*, *w*_max_, and *w*_min_ are the inertia weight, upper and lower bounds of inertia weight, respectively.

### 3.2. Verification and Discussion

**Figure 5:**
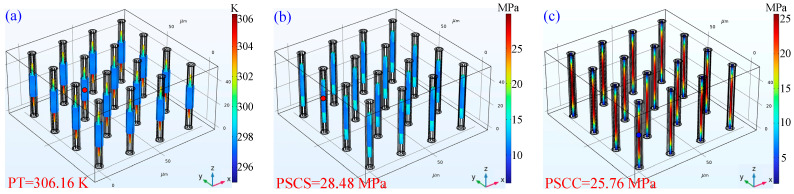
Verified results of FEM model at the optimized parameters: (**a**) PT; (**b**) PSCS; (**c**) PSCC.

**Figure 6:**
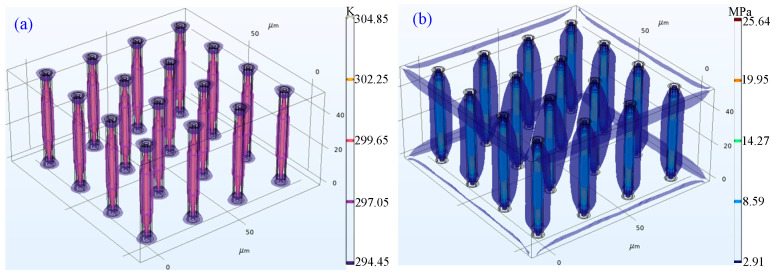
Contour surface of temperature and stress of the verified results: (**a**) temperature; (**b**) stress.

In this research, the efficient thermal-stress coupling design method of CTSV arrays is applied to optimize the structural parameters of CTSV arrays. According to the optimization algorithm, the optimized *r*, *t*_d_, *r*_Cu_, *t*_1_, *t*_2_, *t*_3_, *d*, and *h* are 1, 1.25, 0.64, 0.22, 0.08, 0.15, 28.19, and 53.92 μm, respectively. The simulation results of the FEM model at the optimized structural parameters are shown in Figure 5. The contour surfaces of temperature and stress of the verified results are shown in Figure 6. Clearly, the temperature is higher in the center of the CTSV unit, and the stress distribution is distorted at the interface of different materials. The verified PT, PSCS, and PSCC are 306.16 K, 28.48 MPa, and 25.76 MPa, respectively, while the desired targets are 310 K, 30 MPa, and 25 MPa. Obviously, the verified results agree well with the desired targets. This is because the established SVM model can accurately characterize the FEM model of the CTSV array applied in a Chiplet-based system.

**Figure 7:**
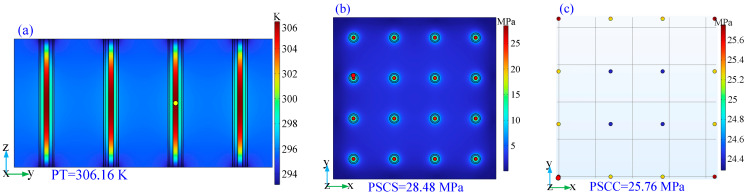
Verified results on the cross section: (**a**) PT; (**b**) PSCS; (**c**) PSCC.

**Figure 8:**
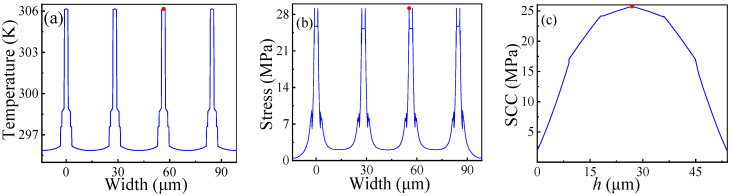
Indexes distribution on the cross section: (**a**) Temperature distribution; (**b**) Stress distribution; (**c**) Stress distribution of copper column (SCC).

In addition, two 2D cross-sections are established to analyze the thermal and stress distribution of the CTSV array. The simulation results are shown in Figure 7. From Figure 7a, the cross-section is x = 28.297 μm. Obviously, the temperature is higher in the center, and the trend of temperature is decreasing from the center to the marginal region. From Figure 7b,c, the cross-section is z = 26.96 μm. The peak stress is located at the interface between different materials. This is because a mismatch in the coefficient of thermal expansion exists between different materials. Moreover, three lines are established to analyze the critical index distribution on the cross section. The simulation results are shown in Figure 8. The temperature curves of the horizontal line (from point (28.297, −14.095, 26.96) to point (28.297, 98.665, 26.96)) are presented in Figure 8a. Clearly, the temperature gradually increases from the edge to the center of the copper column. The stress curves of the horizontal line (from point (0, −14.095, 26.96) to point (0, 98.665, 26.96)) and vertical line (from point (−0.233, −0.287, 0) to point (−0.233, −0.287, 53.92)) are presented in Figure 8b,c. Due to the mismatch of coefficients of thermal expansion between different materials, the stress distribution is buried at the interface of different materials, and the PSCC is located at approximately *h*/2.

The critical indexes of desired, predicted, and verified CTSV arrays are presented in Table 5. The relative errors between the verified and desired targets are all less than 5.07%, while the relative errors between the predicted and desired targets are all less than 2.08%. Clearly, the error exists in the established SVM model, and the computation accuracy of the FEM model is higher than that of the SVM model. In addition, the verified results (306.16 K, 28.48 MPa, and 25.76 MPa) well agree with the desired targets (310 K, 30 MPa, and 25 MPa). Thus, a CTSV array with controllable thermal stress distribution can be obtained by the developed method.

In this research, the CPU of the workstation is Intel(R) Xeon(R) Gold 6242R. The average run time of the SVM model based on MATLAB R2022b is less than 0.05 s, while the average run time of the finite element model based on COMSOL Multiphysics 6.0 is 404 s. Evidently, the FEM simulation is time-consuming and inefficient. In order to increase the simulation efficiency, the SVM is established to characterize the FEM model. In addition, the PSO-LDIW algorithm is adopted to obtain the optimal structural parameters to control the critical indexes of the CTSV array. The average run time of the thermal-stress coupling design based on the SVM model and PSO-LDIW algorithm for one independent run is less than 210 s, while the design cycle of the traditional FEM method is several days to a month for different designers. Thus, the design efficiency of the developed method for CTSV arrays is greatly increased compared with the traditional method. In the future, the placement and routing of Chiplet-based systems with CTSV arrays will be investigated. The range of structural parameters will be expanded to investigate the error of the SVM model, and the dataset will be divided into the training dataset and the testing dataset. The application of the thermal-stress coupling design to more types of TSV structures and more complex 3D integrated circuits with multi-layer structures will also be investigated.

## 4. Conclusions

In this research, an efficient thermal-stress coupling design method for Chiplet-based systems with CTSV arrays is developed based on the SVM model and PSO-LDIW algorithm, which can control the critical indexes of CTSV arrays. The conclusions are summarized as follows:

(1) The relationship between structural parameters and critical indexes of the CTSV array is analyzed based on the established CTSV array model, and it is irregular and complex;
(2) The SVM model is utilized to characterize the complex and irregular relationship between structural parameters and critical indexes of the CTSV array, and the average run time of the SVM model is less than 0.05 s;
(3) The critical indexes of the CTSV array applied in Chiplet-based systems can be controlled by the developed method. The verified results (306.16 K, 28.48 MPa, and 25.76 MPa) well agree with the desired targets (310 K, 30 MPa, and 25 MPa). The average run time of the method for one independent run is less than 210 s, which is much less than the conventional method.

## Figures and Tables

**Table 1:** Physical parameters of material used in the FEM model.

Property	Copper	Si	SiO_2_
Relative permittivity	1	11.7	4.2
Heat capacity at constant pressure (J/(kg·K))	385	700	730
Coefficient of thermal expansion (1/K) × 10^6^	17	2.6	0.5
Density (kg/m^3^)	8960	2329	2200
Thermal conductivity (W/(m·K))	400	130	1.4
Young’s modulus (GPa)	170	170	70
Poisson’s ratio	0.28	0.28	0.17

**Table 2:** Orthogonal experiment.

Factors	1	2	3	4	5	6	7	8	9
*r*	1	1.2	1.5	1.8	2	2.2	2.5	2.8	3
*t*_d_	0.5	0.6	0.7	0.8	1	1.1	1.3	1.4	1.5
*r*_Cu_	0.5	0.6	0.7	0.8	1	1.1	1.3	1.4	1.5
*t*_1_	0.05	0.08	0.1	0.13	0.15	0.18	0.2	0.22	0.25
*t*_2_	0.05	0.08	0.1	0.13	0.15	0.18	0.2	0.22	0.25
*t*_3_	0.05	0.08	0.1	0.13	0.15	0.18	0.2	0.22	0.25
*d*	15	17	20	22	25	28	30	33	35
*h*	40	45	50	55	60	65	70	75	80

**Table 3:** Testing data for the established SVM model.

*r*	*t*_d_	*r*_Cu_	*t*_1_	*t*_2_	*t*_3_	*d*	*h*	FEM			SVM			Maximum Relative Error %
								PT (K)	PSCS (MPa)	PSCC (MPa)	PT (K)	PSCS (MPa)	PSCC (MPa)	
2	0.8	1.1	0.1	0.15	0.15	35	60	319.55	71.36	45.54	322.09	68.71	43.35	5.05
2	1.3	1.1	0.1	0.15	0.15	30	60	316.87	64.44	40.57	322.1	64.73	40.47	1.62
2	0.8	1.1	0.1	0.15	0.15	35	60	318.7	69.01	43.99	322.09	68.71	43.35	1.05
1.8	1	1.1	0.15	0.13	0.15	30	60	317.5	61.88	43.47	322.1	60.8	41.52	4.69
1.8	1	1.1	0.13	0.15	0.18	35	65	318.18	64.68	44.80	322.09	66.95	44.44	1.21
1.8	1	1.1	0.13	0.15	0.15	30	60	317.11	62.10	42.55	322	61.6	40.8	4.29

**Table 4:** Parameters of the efficient thermal-stress coupling design of CTSV array.

Parameters of Optimization Criteria	Desired Critical Indexes	*PT*_des_ = 310 K, *PSCS*_des_ = 30 MPa, *PSCC*_des_ = 25 MPa
Parameters of PSO-LDIW algorithm	Constant parameters	*c*_1_ = *c*_2_ = 2
	Range of inertia weight	$w\in \left[0.4,0.9\right]$
	Maximum generation	*t*_max_ = 100
	Dimension of search space	*D* = 8
	Population size	*N* = 400
	Range of particle position	$\begin{array}{l} x_{r}\in \left[1,3\right],x_{t_{1}{,t}_{2}{.t}_{3}}\in \left[0.05,0.25\right],x_{t_{d}{,r}_{\mathrm{Cu}}}\in \left[0.5,1.5\right]\\ x_{d}\in \left[15,35\right],x_{h}\in \left[40,80\right] \end{array}$
	Range of particle velocity	$\begin{array}{l} v_{r}\in \left[-1,1\right],v_{t_{1}{,t}_{2}{,t}_{3}}\in \left[-0.1,0.1\right],v_{t_{d}{,r}_{\mathrm{Cu}}}\in \left[-0.5,0.5\right]\\ v_{d}\in \left[-1,1\right],v_{h}\in \left[-5,5\right] \end{array}$

**Table 5:** Critical indexes of CTSV array at the optimized parameters.

Indexes	PT (K)	PSCS (MPa)	PSCC (MPa)
Desired	310	30	25
Predicted	310	29.52	25.52
Verified	306.16	28.48	25.76

## Data Availability

Not applicable.
